# Circular Valorization of Laurel and Myrtle Pruning Residues: Chemical Profiling, Phytotoxicity, and Ecotoxicological Assessment of Essential Oils for Sustainable Weed Management

**DOI:** 10.3390/molecules31162741

**Published:** 2026-08-07

**Authors:** Flavio Polito, Paola Malaspina, Annarita La Neve, Antonella Smeriglio, Laura Cornara, Vincenzo De Feo, Domenico Trombetta

**Affiliations:** 1 Department of Pharmacy, University of Salerno, Via Giovanni Paolo II 132, 84084 Fisciano, Italy; fpolito@unisa.it (F.P.); defeo@unisa.it (V.D.F.); 2 Department of Earth, Environment and Life Sciences, University of Genova, Corso Europa 26, 16132 Genova, Italy; paola.malaspina@unige.it; 3 Department of Chemical, Biological, Pharmaceutical and Environmental Sciences (ChiBioFarAm), University of Messina, 98166 Messina, Italy; annarita.laneve@unime.it (A.L.N.); domenico.trombetta@unime.it (D.T.)

**Keywords:** *Laurus nobilis* L., *Myrtus communis* L., secretory tissues, seed germination, phytochemistry, ecotoxicology, bioherbicides

## Abstract

The valorization of medicinal and aromatic plant by-products represents a sustainable strategy to recover bioactive compounds and reduce agricultural waste. In this study, pruning residues of *Laurus nobilis* L. and *Myrtus communis* L. were investigated as sources of essential oils (EOs) for bio-based weed management. Leaves and young stems were subjected to anatomical, micromorphological, and histochemical analyses to ascertain that active secretory structures were maintained. EOs were then obtained by steam distillation and characterized by GC-FID and GC-MS. Phytotoxicity was evaluated on two crop species, *Hordeum vulgare* L. and *Raphanus sativus* L., and two weed species, *Lolium multiflorum* Lam. and *Sinapis alba* L.; ecotoxicity was assessed using *Artemia salina* and *Daphnia magna*. The EOs were mainly composed of oxygenated monoterpenes, with 1,8-cineole as the dominant constituent in both oils. Phytotoxicity was species- and concentration-dependent, with *M. communis* EO showing the strongest inhibition, particularly against *S. alba*. Ecotoxicological assays revealed limited toxicity toward *A. salina*, but concentration-dependent immobilization of *D. magna*. Overall, these findings support the circular valorization of laurel and myrtle pruning residues as sources of bioactive EOs while highlighting the need for further formulation, selectivity, and environmental safety studies before agricultural application.

## 1. Introduction

Within the research and innovation priorities of the National Strategic Plan for the Development of the Organic System [1], the recovery of plant by-products represents an important objective, as it can significantly contribute to waste reduction and to the development of circular economy strategies based on the efficient use of renewable biological resources [2]. In this context, a new perspective is represented by the recovery of by-products resulting from the cultivation, pruning, and processing of medicinal and aromatic plants (MAPs), which constitute a constantly expanding supply chain in Europe and globally, driven by an annual growth rate of about 10% in the phytotherapeutic and biocosmetic sectors [3]. Furthermore, this sector also represents a source of significant economic growth in Europe due to the multiple applications of MAP-derived products in food processing and production [4,5].

Therefore, given the significant biomass generated annually from pruning and processing of MAPs, the valorization of this waste for extracting bioactive compounds opens up promising applications, such as the development of effective bioherbicides for weed management. Indeed, leaves and young branches discarded after pruning may still contain specialized secretory structures rich in volatile bioactive compounds, making them suitable raw materials for the recovery of high-value phytochemicals through low-impact approaches [2,6]. These plant complexes can support sustainable agriculture by drastically reducing reliance on synthetic chemicals, which are harmful to both the environment and human health. Among these compounds, essential oil (EO) terpenes exhibit strong allelochemical properties, making them viable alternatives to synthetic herbicides while avoiding their negative side effects [7,8]. In particular, several EO constituents, mainly mono- and sesquiterpenes, may interfere with seed germination, radicle elongation, membrane integrity, oxidative balance, and early seedling development, supporting their potential use as bioherbicidal agents [9,10]. However, despite their natural origin, EOs cannot be automatically considered environmentally safe. Their biological activity, volatility, chemical complexity, and potential toxicity to non-target organisms require an integrated assessment that combines chemical profiling, phytotoxic evaluation, and preliminary ecotoxicological screening [11]. With this objective in mind, our research focused on two aromatic species, *Laurus nobilis* L. and *Myrtus communis* L., which are widely cultivated in the Mediterranean region as potted plants and also extensively used for landscaping and ornamental hedges.

In addition to the wide use of laurel EO as a food additive and flavoring agent, and for different medicinal and health-promoting applications [12], recent research has also explored its promising properties for sustainable agriculture. For example, Boukabache et al. [13] have shown that this EO and its main compound, 1,8-cineole, possess high aphicide activity, paving the way for the development of innovative plant-based insecticides for the management of agricultural pests. Regarding the potential application of laurel EO as a bioherbicide, Hazrati et al. [14] studied its efficacy both pure and in combination with rosemary EO. When applied alone, laurel EO showed modest phytotoxic activity on the germination of weeds such as *Amaranthus retroflexus* L., which, however, was increased when combined with rosemary EO. Furthermore, the presence of laurel in the mixture reduced the marked phytotoxicity that rosemary EO exerted on tomato germination, carrying out a mitigating and protective action on the agricultural crop. During post-emergence growth, however, the laurel–rosemary combination showed a clear synergistic effect, causing a greater inhibition of the development of *A. retroflexus* than that induced by the two EOs applied individually. In a more recent study, Tursun et al. [15] reported that laurel EO and its main component, 1,8-cineole, showed a significant difference in the response of the tested weed species, but had little selective action on the crop species examined.

The EO of *M. communis* L. is mainly employed in the flavor and perfume industries. In addition, it contains a vast array of pharmacologically active compounds with antimicrobial, insecticidal, antioxidant, and hepatoprotective properties [16,17]. Recent studies have also begun to evaluate the biological activities of myrtle EO for agricultural applications. Benddine et al. [18] showed the insecticidal properties of myrtle EO, dominated by α-pinene (55.47%) and 1,8-cineole (28%), on heterogeneous populations of *Rhopalosiphum maidis*, a pest of corn crop, tested under field conditions. Miloudi et al. [19], while assessing the phytotoxicity of different EOs, determined that a mixture of 22% of *M. communis*, 23% of *Satureja alpina* L., and 55% of *Thymus satureioides* Coss was optimal against the weed species *Amaranthus retroflexus* L. Nevertheless, available data on the phytotoxic potential of myrtle EO remain limited, and its selectivity toward crops and weeds requires further investigation.

Therefore, considering that data on the potential phytotoxic effects of the two EOs of laurel and myrtle are scarce and controversial, we decided to investigate this issue further. The pruning waste obtained from evergreen hedges was first analyzed to locate the secretory structures and ensure that they were still rich in EO. The two EOs were then distilled, chemically characterized, and evaluated for their phytotoxic and ecotoxicological effects in order to assess their potential as bio-based weed management agents and the possible environmental risks to non-target aquatic organisms associated with their use [11]. The collected data could provide the baseline requirements for formulating new natural herbicides suitable for organic farming systems.

The novelty of the present study lies in the integrated valorization of pruning residues through anatomical, micromorphological, and histochemical characterization of the plant material, combined with phytochemical profiling and phytotoxic and ecotoxicological assessment of the resulting EOs.

## 2. Results

### 2.1. Anatomy and Micromorphology

**Figure 1:**
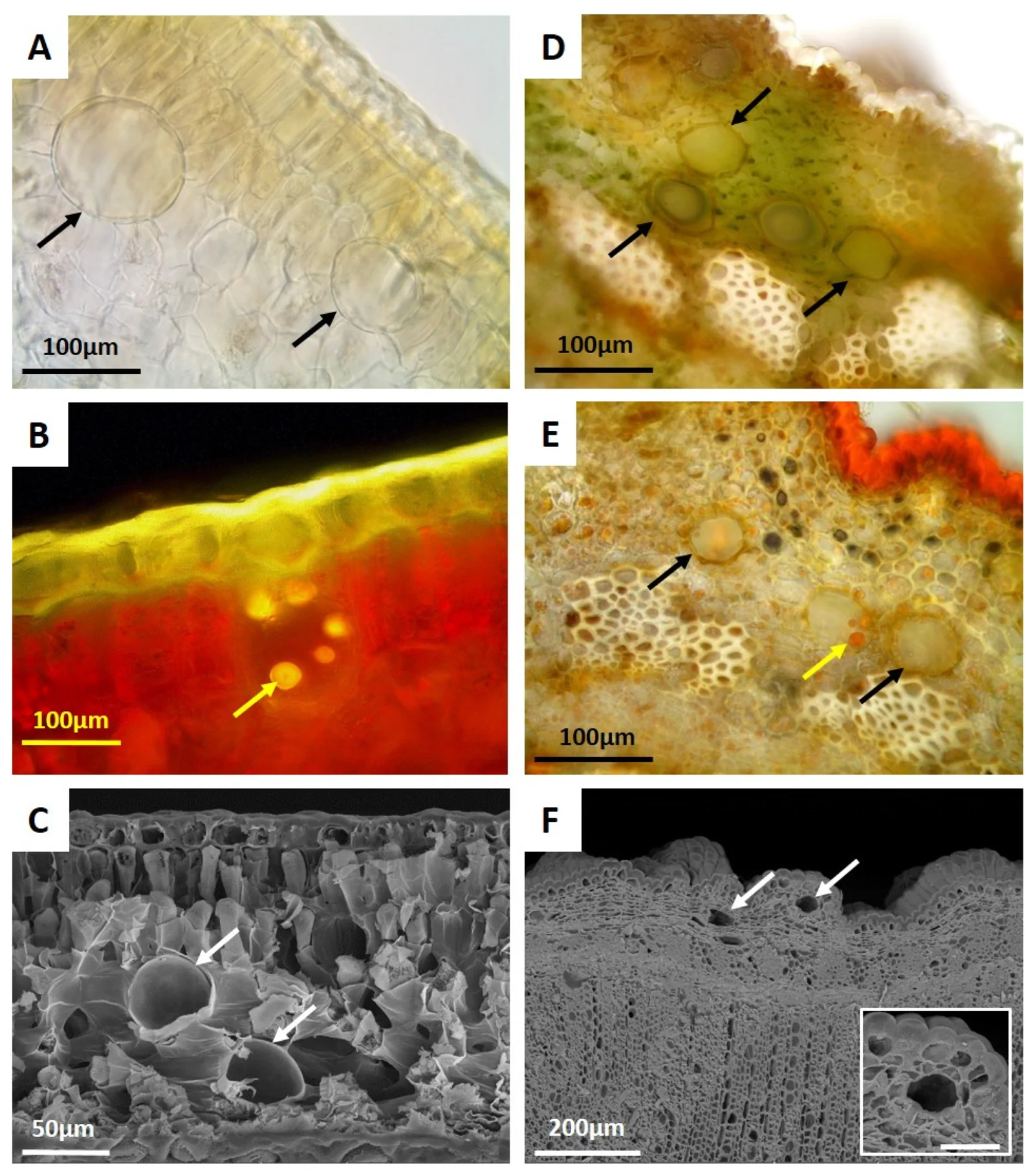
*L. nobilis* transverse sections of the leaf (**A**–**C**) and young stem (**D**–**F**). Light Microscopy (**A**,**B**,**D**,**E**) and Scanning Electron Microscopy (**C**,**F**). Many large spheroidal secretory idioblasts are visible both in the leaf mesophyll (**A**–**C**, arrows) and in the stem cortex (**D**–**F**, arrows); the insert in (**F**) shows an enlargement of a single idioblast. EO within the idioblasts is shown by the positive reaction with both Fluorol Yellow 088 (**B**, arrow) and Sudan III (**E**, yellow arrow).

In *L. nobilis*, several secretory idioblasts were observed throughout the leaf mesophyll and were distinguished from the neighboring cells in their larger size and subspherical shape (Figure 1A–C, arrows). In young stems, numerous large idioblasts were also detected, mainly localized in the cortical zone (Figure 1D–F, arrows), between the phloem sclerenchymatic fibers and the epidermis. The latter was covered by a thick cuticle that reacted positively with Sudan III (Figure 1E). Within the secretory idioblasts, the presence of EO was detected by both Fluorol Yellow 088 (Figure 1B, arrow) and Sudan III (Figure 1E, yellow arrow).

**Figure 2:**
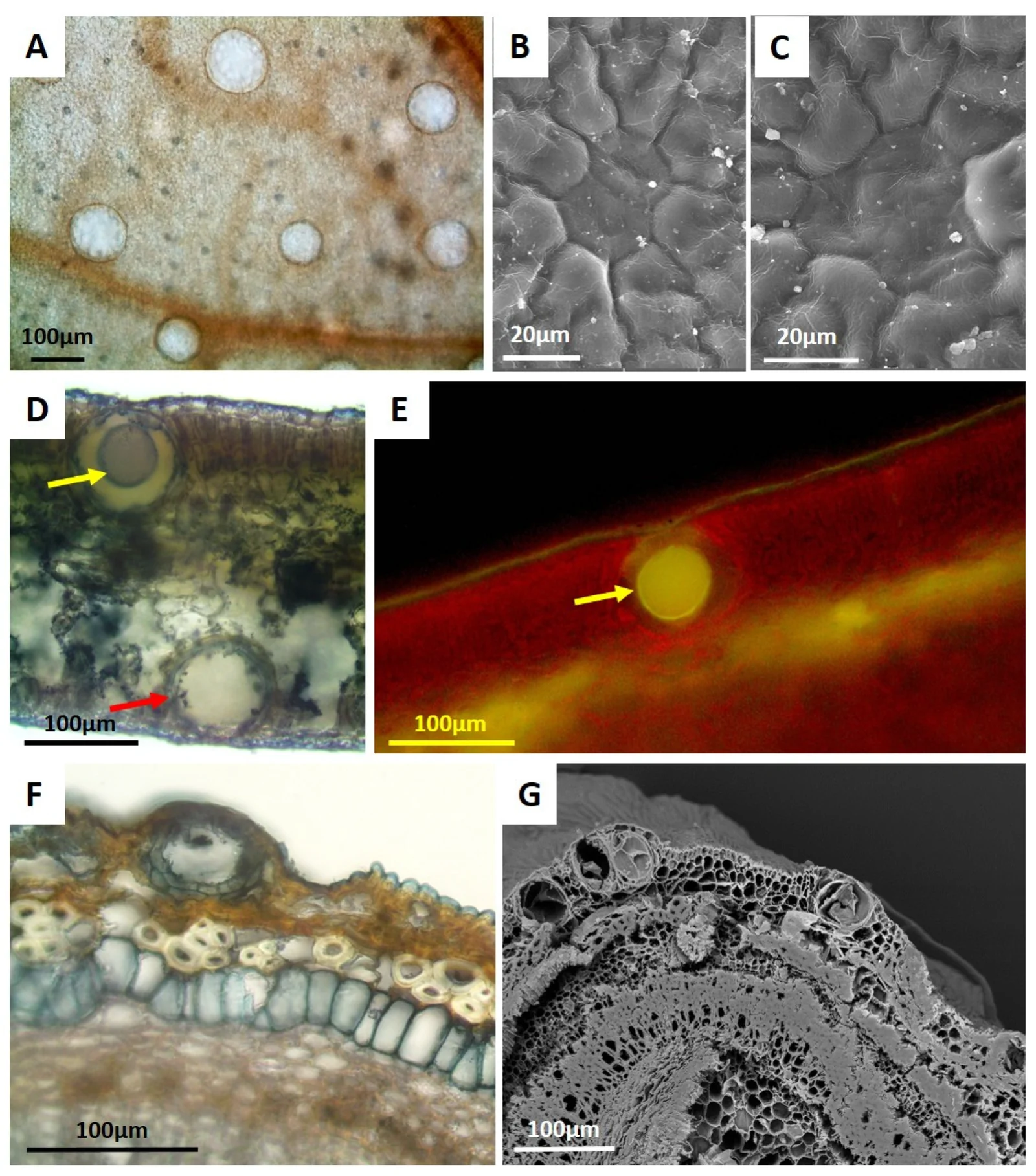
Light Microscopy (**A**,**D**,**E**,**F**) and Scanning Electron Microscopy (**B**,**C**,**G**) of *M. communis* leaves (**A**–**E**) and young stem (**F**,**G**). Numerous secretory cavities are visible on the foliar epidermal surface (**A**); SEM details of the overlying cells, showing groups of two (B) or three (C) cells; leaf transverse sections stained with Sudan Black (**D**) and Fluorol Yellow 088 (**E**). The yellow arrows indicate the EO droplets that positively reacted with the histochemical stains, and the red arrow indicates the single epithelial layer of secretory cells. A stem transverse section stained with Sudan Black (**F**); several secretory cavities are visible in the outer layers of the cortex parenchyma, under the epidermis (**G**).

The occurrence of secretory cavities in leaves and young stems of *M. communis* was shown by light (LM) and scanning electron microscopy (SEM) (Figure 2A–G). Foliar epidermal surfaces, bleached with a chloral hydrate solution and observed by LM, allowed the detection of numerous secretory cavities, which were large and spherical in shape (Figure 2A). A more in-depth analysis with SEM highlighted the presence of two (Figure 2B) or three (Figure 2C) overlying cells located above the secretory cavities. In handmade transversal sections, we observed that the secretory cavities, scattered in the leaf mesophyll, were mainly located near the subepidermal region on both leaf surfaces. The cavities showed a spherical shape and displayed a single epithelial layer of secretory cells (Figure 2D, red arrow) that released EO into the cavity (Figure 2D,E, yellow arrows). Numerous secretory cavities were also found in the young stems, mainly localized in the outer layers of the cortex parenchyma, under the epidermis (Figure 2F,G). Histochemical analysis showed comparable results for the secretory cavities of both leaves (Figure 2D,E) and young stems (Figure 2F). In particular, positive reactions were observed with both Sudan Black, which yielded dark-blue staining (Figure 2D,F), and Fluorol Yellow 088, which produced a bright yellow fluorescence (Figure 2E), indicating the occurrence of lipophilic compounds in the EO.

### 2.2. Essential Oils Yield and Chemical Composition

The percentage yields of EOs were 0.42% for *L. nobilis* and 0.26% for *M. communis*. Analyses performed by gas chromatography with flame ionization detection (GC-FID) and gas chromatography–mass spectrometry (GC-MS) allowed the identification of all the compounds present in the EOs, accounting for 100% of the total detected composition.

Thirteen compounds were identified in *L. nobilis* EO, as reported in Table 1. Most of them belonged to the oxygenated monoterpene class (69.05%), followed by hydrocarbon monoterpenes (26.55%) and phenylpropanoids (4.40%). The most abundant compounds were 1,8-cineole (46.31%), sabinene (16.05%), and linalool (13.67%). Additionally, α-pinene and β-terpinyl acetate were present in amounts greater than 5% (6.96% and 5.68%, respectively), while all other compounds were detected at levels below 5%.

Table 2 reports the composition of *M. communis* EO. Among the 21 identified compounds, oxygenated monoterpenes represented the predominant class (74.72%), followed by hydrocarbon monoterpenes (23.11%). Phenylpropanoids and hydrocarbon sesquiterpenes were present in lower amounts (1.10% and 1.07%, respectively). The main constituents were 1,8-cineole (41.53%), α-pinene (18.43%), and myrtenyl acetate (16.06%). All other compounds, except for linalool (8.51%), were present at levels below 5%.

### 2.3. Phytotoxic Activity

Table 3 and Table 4 show the phytotoxic activity of the tested EOs on the germination and radicle elongation of four selected species: the two crops *H. vulgare* and *R. sativus*, and the two weeds *L. multiflorum* and *S. alba*. The tables report the mean values, standard deviations, and statistical significance for germination (number of germinated seeds) and radicle elongation (cm) measurements. Percentage inhibition values, calculated relative to the control solution consisting of water and acetone (99.5:0.5 *v*/*v*), are reported in parentheses next to each value. The control treatment was assigned an inhibition value of 0.0%. These percentages were included only to facilitate the visualization and interpretation of the phytotoxic effects, whereas statistical analyses were performed on the original datasets. In each table, positive inhibition values (highlighted in green) indicate a reduction in germination or radicle elongation, whereas negative values (highlighted in red) indicate a stimulatory effect compared with the control. The intensity of the color reflects the magnitude of the response. White color indicates 0.0% inhibition.

**Figure 3:**
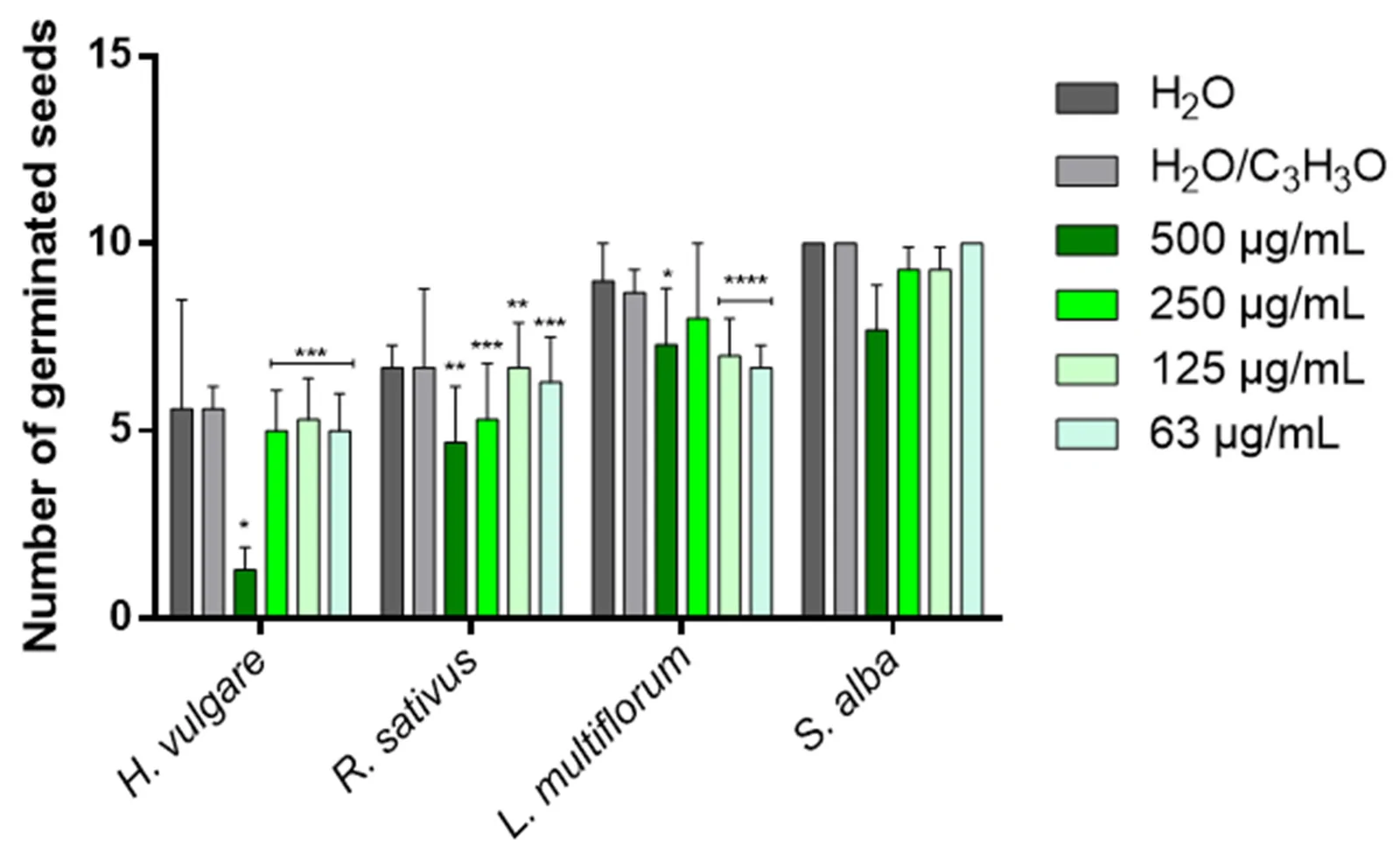
Bar graph of the phytotoxic activity of the *L. nobilis* EO against the germination of *H. vulgare*, *R. sativus*, *L. multiflorum,* and *S. alba*. The results are reported as the mean of three experiments ± the standard deviation. * *p* < 0.05; ** *p* < 0.01; *** *p* < 0.001; **** *p* < 0.0001 compared with control (ANOVA followed by Dunnett’s multiple-comparison test).

**Figure 4:**
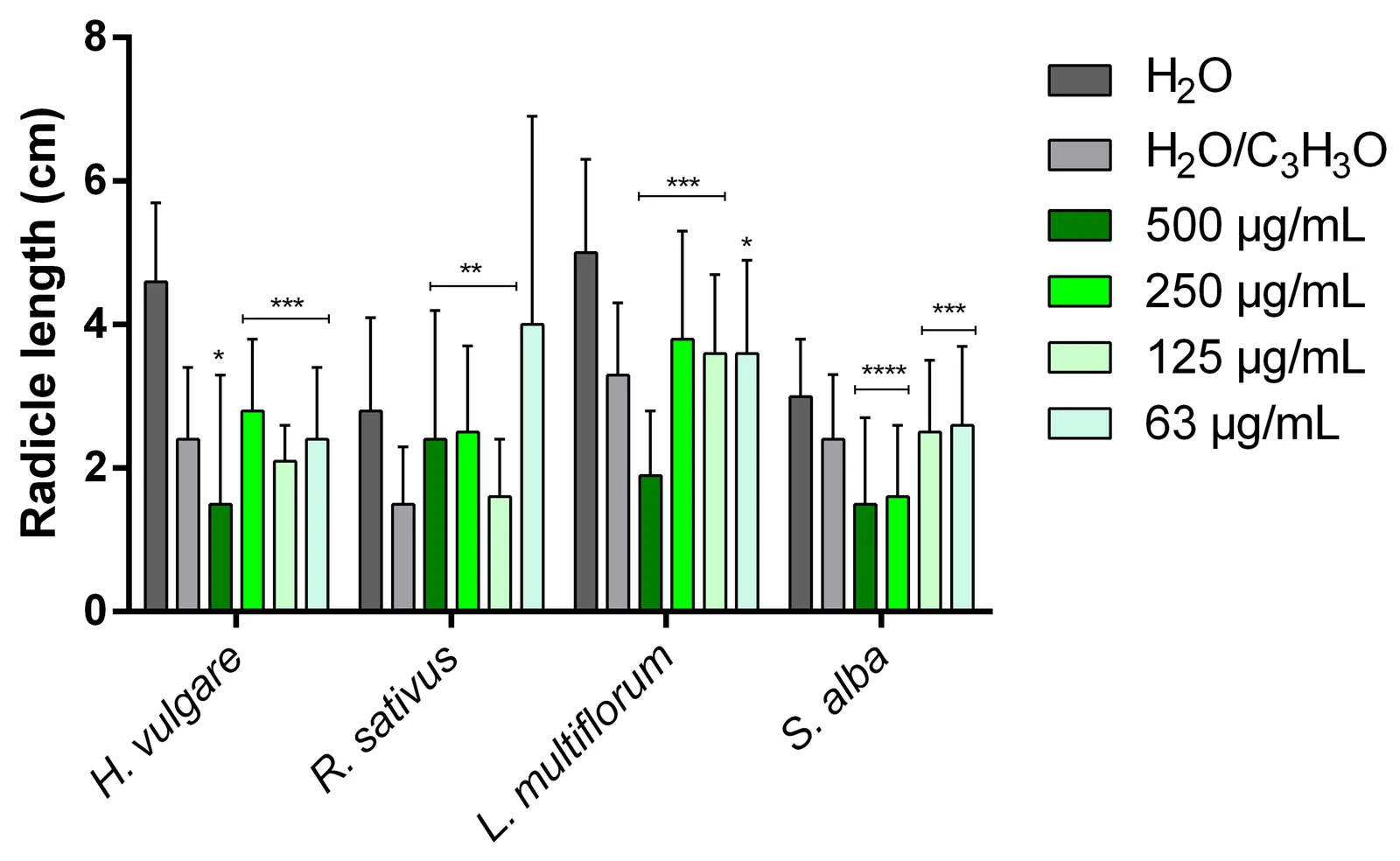
Bar graph of the phytotoxic activity of the *L. nobilis* EO against the radicle elongation of *H. vulgare*, *R. sativus*, *L. multiflorum,* and *S. alba*. The results are reported as the mean of three experiments ± the standard deviation. * *p* < 0.05; ** *p* < 0.01; *** *p* < 0.001; **** *p* < 0.0001 compared with control (ANOVA followed by Dunnett’s multiple-comparison test).

**Figure 5:**
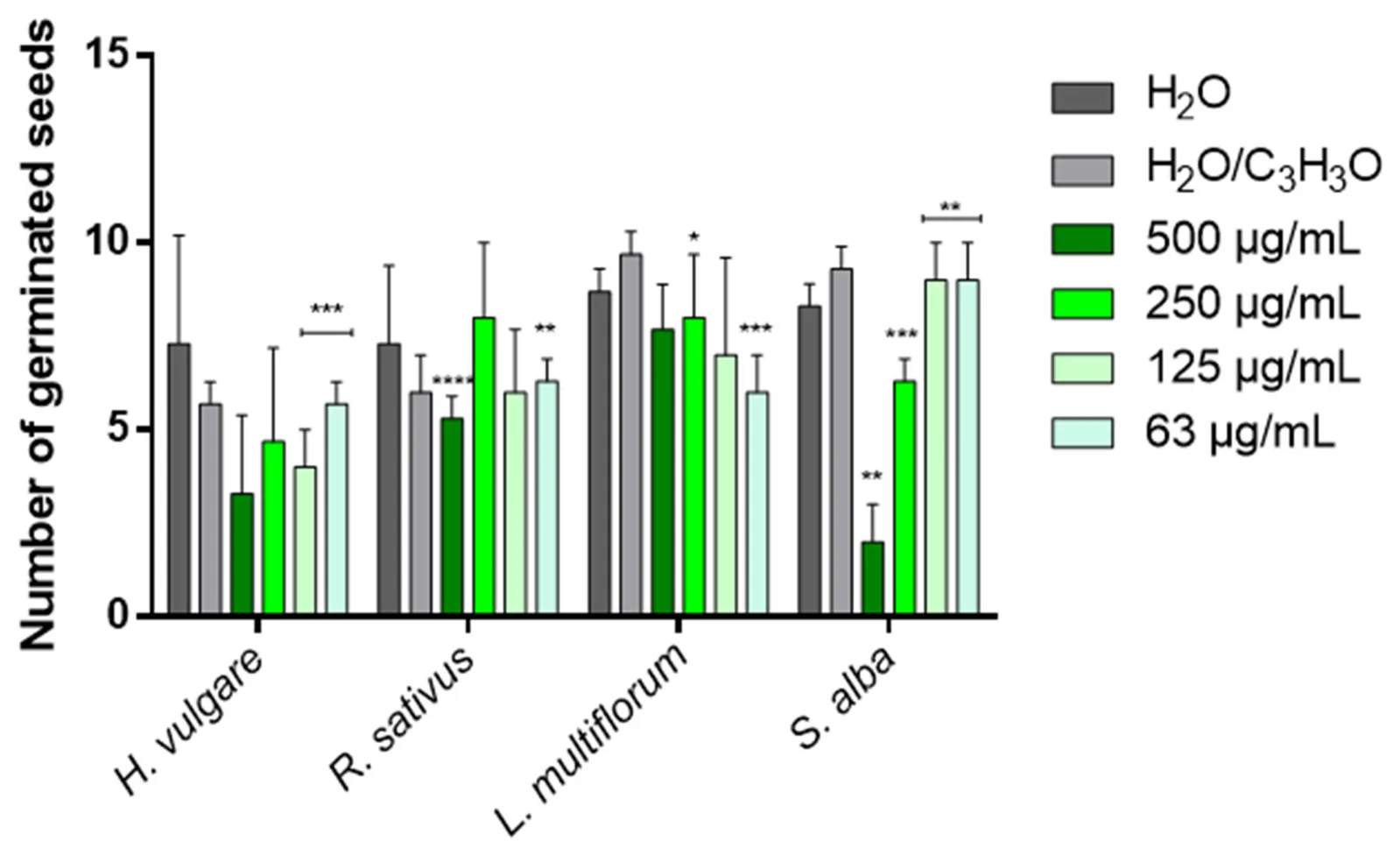
Bar graph of the phytotoxic activity of the *M. communis* EO against the germination of *H. vulgare*, *R. sativus*, *L. multiflorum,* and *S. alba*. The results are reported as the mean of three experiments ± the standard deviation. * *p* < 0.05; ** *p* < 0.01; *** *p* < 0.001; **** *p* < 0.0001 compared with control (ANOVA followed by Dunnett’s multiple-comparison test).

**Figure 6:**
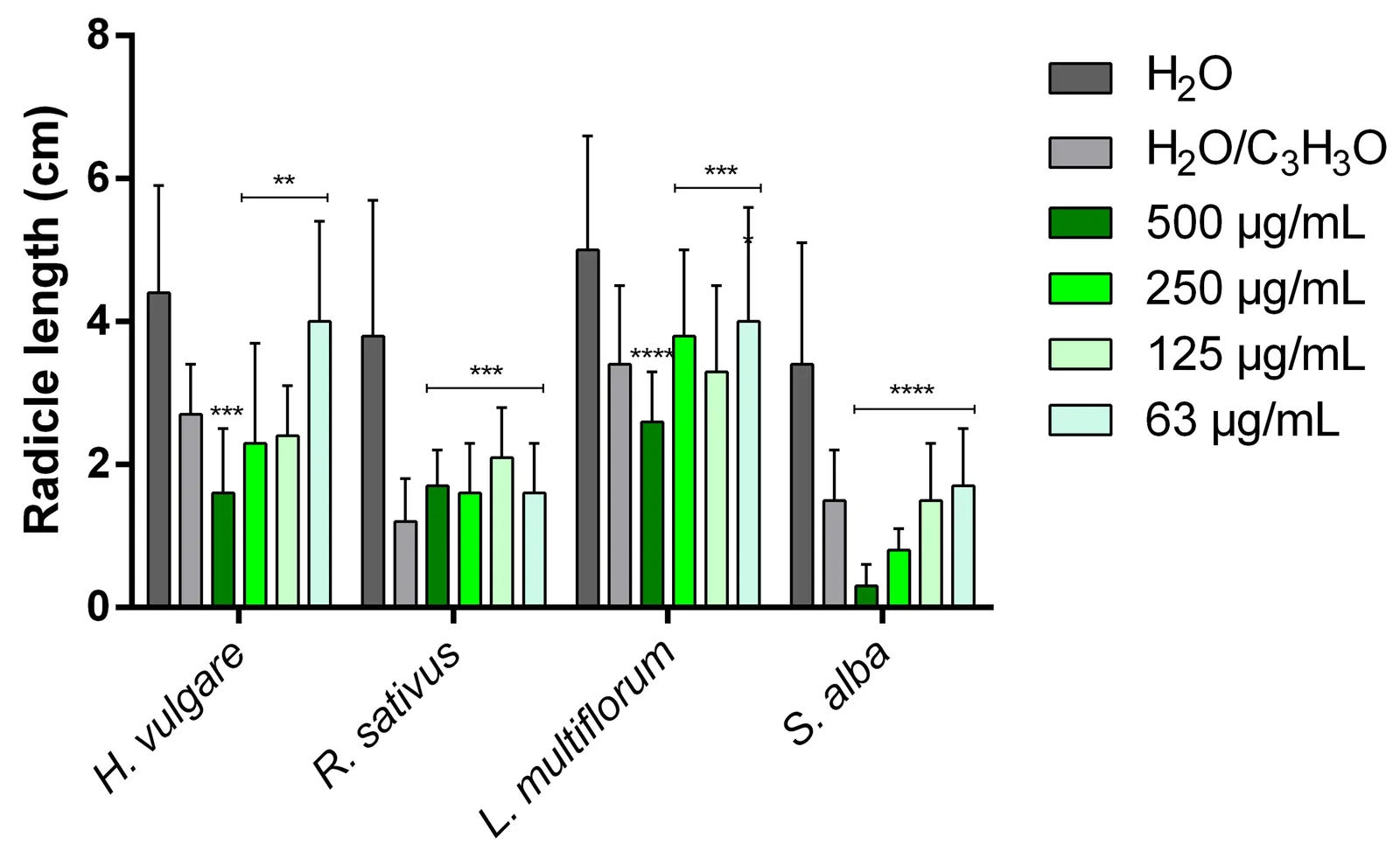
Bar graph of the phytotoxic activity of the *M. communis* EO against the radicle elongation of *H. vulgare*, *R. sativus*, *L. multiflorum,* and *S. alba*. The results are reported as the mean of three experiments ± the standard deviation. ** *p* < 0.01; *** *p* < 0.001; **** *p* < 0.0001 compared with control (ANOVA followed by Dunnett’s multiple-comparison test).

Figure 3, Figure 4, Figure 5 and Figure 6 show bar graphs used to describe the same effects of the EO solutions on the germination and radicle elongation. The graphs were generated from the original data on the number of germinated seeds and radicle length.

Overall, the EOs showed concentration-dependent and species-specific phytotoxic effects, with different responses depending on the plant species and the endpoint considered. In general, the highest concentrations produced the most evident inhibitory effects, whereas lower concentrations induced weaker inhibition or, in some cases, stimulation of radicle elongation. The EO from *L. nobilis* was generally more active on the germination of *H. vulgare* and *L. multiflorum*, and on the radicle elongation of *L. multiflorum* and *S. alba*. In *H. vulgare*, germination was only slightly affected at lower concentrations, whereas the highest concentration (500 µg/mL) caused a marked inhibition of 76.8%. Radicle elongation showed a more heterogeneous response, with modest inhibition at 125 and 500 µg/mL and slight stimulation at 250 µg/mL. In *R. sativus*, germination was only modestly inhibited at the two highest concentrations, while radicle elongation was stimulated at all tested concentrations. In *L. multiflorum*, germination was modestly inhibited across almost all concentrations, whereas radicle elongation was inhibited only at 500 µg/mL. In *S. alba*, the germination process was affected mainly at the highest concentration, while radicle elongation was inhibited at the two highest concentrations and slightly stimulated at the lower ones. The EO from *M. communis* showed more pronounced phytotoxic effects, particularly against *S. alba*. In *H. vulgare*, both germination and radicle elongation were moderately inhibited at most concentrations, although the lowest concentration stimulated radicle elongation. In *R. sativus*, germination was only weakly affected, whereas radicle elongation was strongly stimulated at all tested concentrations. In *L. multiflorum*, germination was moderately inhibited, whereas radicle elongation showed a concentration-dependent and heterogeneous response, with stimulation at some lower concentrations and inhibition at 500 µg/mL. *S. alba* was the most sensitive species to *M. communis* EO, showing strong inhibition of both germination and radicle elongation at the highest concentration, with maximum inhibition values of 78.5% and 80.0%, respectively. Overall, the two EOs showed species- and concentration-dependent phytotoxic effects, with some weed species displaying greater sensitivity than the tested crop species depending on the endpoint evaluated. Among the crop species, *H. vulgare* was moderately affected, whereas *R. sativus* appeared more tolerant and often showed stimulation of radicle elongation. These results suggest the potential use of these EOs as candidate bio-based tools for weed management, although further selectivity and application studies are required.

### 2.4. Ecotoxicological Effects

The ecotoxicological assessment of the EOs of *L. nobilis* and *M. communis* was carried out using two aquatic organisms representative of different ecosystems: *Artemia salina*, as a marine/brackish-water model, and *Daphnia magna*, as a freshwater model.

**Figure 7:**
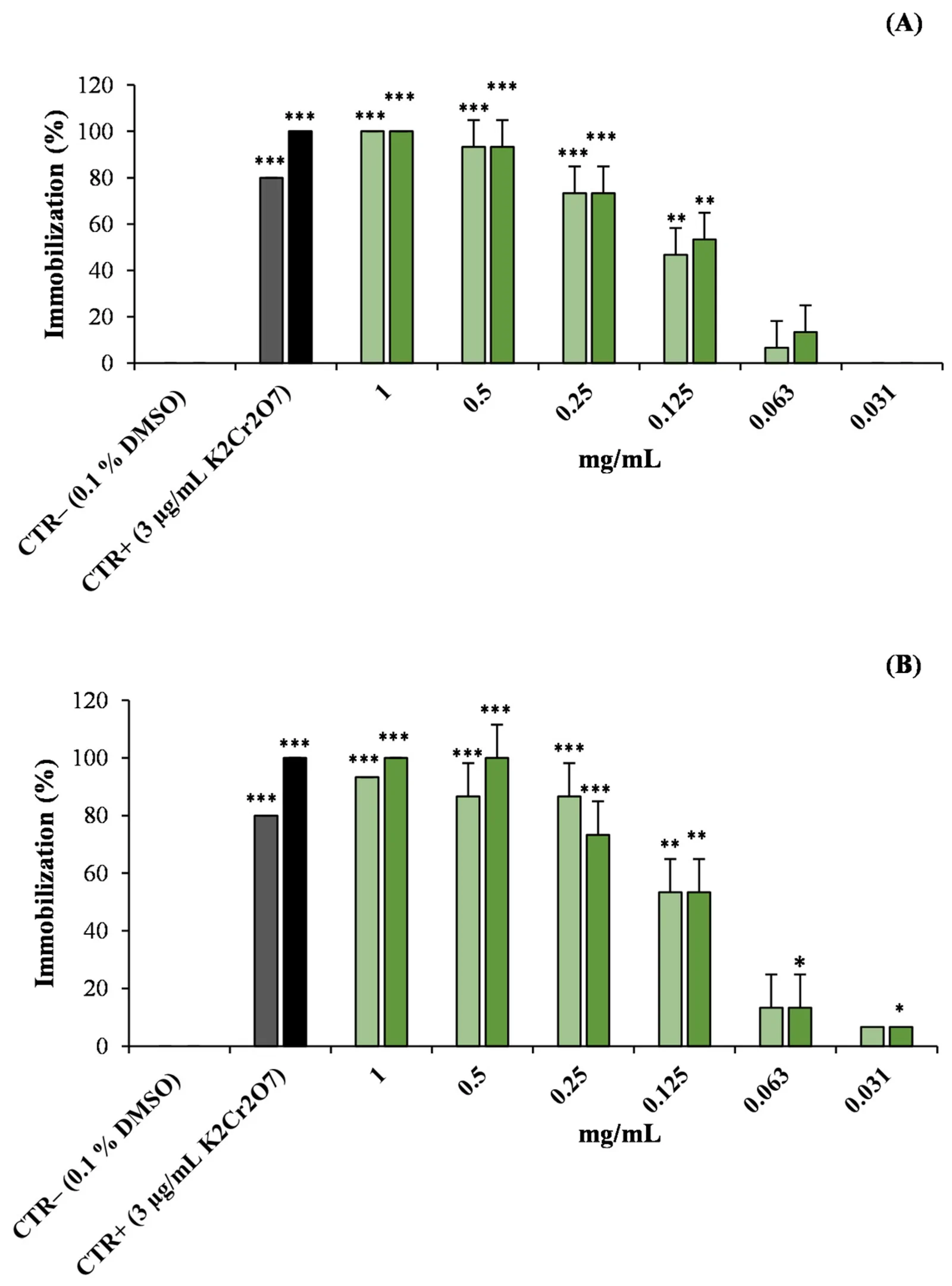
Immobilization of *D. magna* neonates after exposure to different concentrations of the essential oils (EOs) of (**A**) *M. communis* L. and (**B**) *L. nobilis* L., after 24 and 48 h of exposure. The negative control (CTR−) bars are not visible at either 24 or 48 h because immobilization was 0% at both time points. Potassium dichromate (K_2_Cr_2_O_7_, 3 µg/mL), is shown in dark grey at 24 h and black at 48 h. For all EO treatments, light green indicates the response at 24 h, whereas dark green indicates the response at 48 h. Data are presented as mean ± standard deviation (SD) of three independent experiments, each performed in triplicate. Dimethyl sulfoxide (DMSO 0.1%) was used as the CTR−, whereas K_2_Cr_2_O_7_, 3 µg/mLwas used as the positive control (CTR+). Statistical significance is reported versus the negative control (DMSO 0.1%): * *p* < 0.05; ** *p* < 0.01; *** *p* < 0.001.

Exposure of *D. magna* to increasing concentrations of the EOs resulted in a statistically significant, concentration-dependent increase in immobilization compared with the negative control. The highest effects were observed at 1 and 0.5 mg/mL, reaching values close to 100% immobilization, while effects progressively decreased at lower concentrations (Figure 7).

For *M. communis* EO (Figure 7A), 1 mg/mL induced complete immobilization after both 24 and 48 h, while at 0.5 mg/mL, an immobilization of approximately 93% was observed at 24 h and confirmed at 48 h. A clear concentration-response pattern was also evident at 0.25 and 0.125 mg/mL, which induced approximately 73% and 47–53% immobilization, respectively, whereas the two lowest concentrations showed only limited or no effects. A similar trend was observed for *L. nobilis* EO (Figure 7B), where 1 mg/mL increased immobilization from approximately 93% at 24 h to 100% at 48 h, whereas 0.5 mg/mL resulted in approximately 87% and 100% immobilization at 24 and 48 h, respectively. At 0.25 mg/mL, immobilization remained high, although a slight decrease was observed at 48 h, while lower concentrations produced moderate or negligible effects. Overall, statistically significant immobilization was mainly observed from 0.125 to 1 mg/mL, whereas the lowest concentrations did not differ significantly from the negative control. Potassium dichromate (K_2_Cr_2_O_7_, 3 µg/mL), used as the positive control, caused a pronounced and statistically significant immobilization of *D. magna* as expected, validating the assay. The median effective concentration (EC_50_) for *D. magna* was 0.155 mg/mL (95% CL: 0.134–0.180 mg/mL) and 0.147 mg/mL (95% CL: 0.128–0.170 mg/mL) after 24 and 48 h exposure to *M. communis* EO, respectively. For *L. nobilis*, EC_50_ values were 0.143 mg/mL (95% CL: 0.08–0.26 mg/mL) at 24 h and 0.12 mg/mL (95% CL: 0.08–0.163 mg/mL) at 48 h.

**Figure 8:**
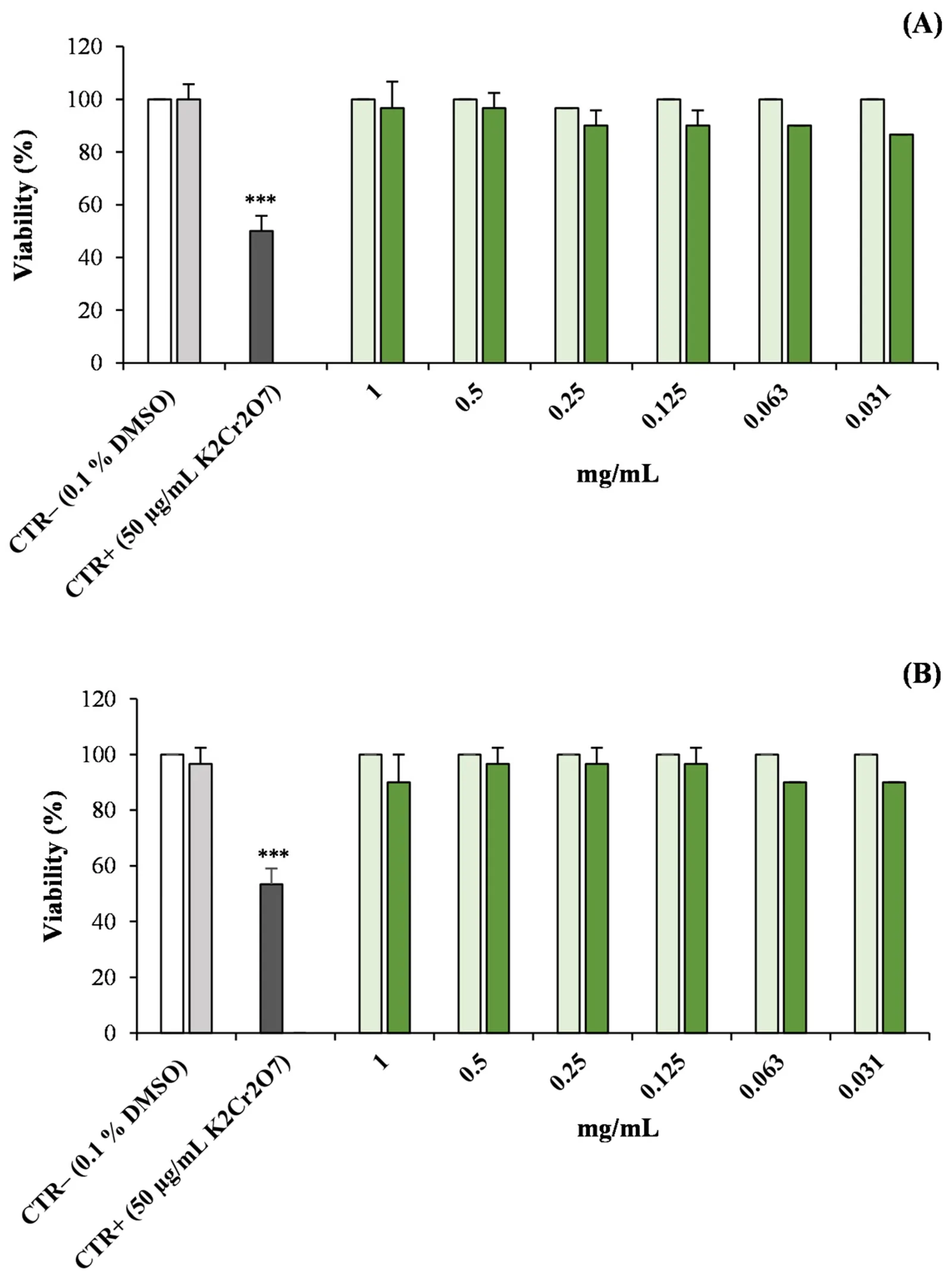
Viability of *A. salina* nauplii after exposure to different concentrations of the essential oils (EOs) of (**A**) *M. communis* L. and (**B**) *L. nobilis* L., after 24 and 48 h of exposure. The negative control (CTR−) is represented in white at 24 h and light grey at 48 h, whereas potassium dichromate (K_2_Cr_2_O_7_, 50 µg/mL) is shown in dark grey at 24 h. The K_2_Cr_2_O_7_ bar is not visible at 48 h because viability was 0% at this time point. For all EO treatments, light green indicates the response at 24 h and dark green indicates the response at 48 h. Data are presented as mean ± standard deviation (SD) of three independent experiments, each performed in triplicate. Dimethyl sulfoxide (DMSO 0.1%) was used as the CTR−, whereas K_2_Cr_2_O_7_, 50 µg/mL was used as the positive control (CTR+). Statistical significance is reported versus the negative control DMSO 0.1%: *** *p* < 0.001.

In contrast, *A. salina* nauplii showed no statistically significant reduction in viability across the tested concentration range (0.031–1 mg/mL) after either 24 or 48 h (Figure 8).

Viability remained comparable to the negative control (DMSO, 0.1%), with no statistically significant differences between the treated and control groups. Although a slight decrease in viability was observed after 48 h at some concentrations, particularly at the lowest tested levels, this effect was not statistically significant and did not indicate a clear concentration-dependent toxicity. As expected, the positive control (K_2_Cr_2_O_7_, 50 μg/mL) caused a marked and significant reduction in viability (*p* < 0.001) compared with the negative control and with the EO-treated groups, confirming the validity of the assay.

Overall, the ecotoxicological response was model-dependent. Both EOs induced a clear concentration-dependent immobilization of *D. magna*, whereas no significant toxicity was observed in *A. salina* under the same exposure conditions. These findings indicate that the environmental safety profile of the tested EOs should be considered organism-specific, highlighting the importance of including freshwater non-target organisms in the preliminary risk assessment of EO-based products.

## 3. Discussion

The sustainable management of plant biomass generated from pruning and processing activities represents a relevant challenge within circular economy and green chemistry strategies. In this context, pruning residues should not be regarded only as agricultural or ornamental waste, but also as renewable matrices potentially rich in specialized metabolites. Recent studies have highlighted the possibility of converting urban and agricultural pruning residues into value-added materials and bioactive products, supporting their inclusion in biorefinery and circular valorization approaches [20,21].

With a view to reusing these valuable compounds, in a previous study, we highlighted that pruning residues from lavender and helichrysum can represent a promising biomass for the recovery of bioactiveplant–complexes with potential applications in sustainable weed management [22].

In the present study, we recovered pruning residues from laurel and myrtle, two aromatic species widely cultivated in the Mediterranean region as potted plants and also extensively used for ornamental hedges. The EOs obtained from these by-products were chemically characterized and evaluated as candidate bio-based tools for weed management, within an integrated approach combining botanical quality control, phytotoxicity, and ecotoxicological assessment. Microscopic analysis proved to be a useful quality-control tool for verifying that the volatile fraction had not been lost from the plant residues because of evaporation or mechanical rupture. Our observations showed that secretory structures containing EOs were preserved in the pruned residues and revealed a high abundance of idioblasts and secretory cavities in the young branches of laurel and myrtle, respectively. These findings suggested that including leafy twigs in the distillation process could increase EO yields. To the best of our knowledge, only a limited number of studies have investigated the anatomical and micromorphological characteristics of both leaves and young stems of *L. nobilis* [23,24,25,26]. Consistent with the findings of these authors, the present micromorphological analysis identified the presence of large secretory oil cells throughout the leaf mesophyll and stem cortical parenchyma as diagnostic features of *L. nobilis*. The occurrence of EO within idioblast cells was confirmed by positive reactions with the lipophilic dyes Sudan III and Fluorol Yellow 088. Furthermore, a thick cuticular layer covering the epidermis was also detected, representing a typical xeromorphic adaptation that contributes to the reduction in transpirational water loss. These anatomical and histochemical traits provide reliable diagnostic markers for the identification and quality assessment of *L. nobilis* residues, even when the plant material is available as fragmented leaves and stems [24].

*M. communis* leaves showed large secretory cavities on adaxial and abaxial surfaces of the leaf, distributed in palisade and spongy tissues. This organization is consistent with previous reports describing secretory cavities as characteristic structures of this species and the Myrtaceae family [27,28,29]. According to previous studies, these cavities are characterized by a single layer of secretory epithelial cells, and the secreted material consists of oil droplets that may fill the cavity lumen [30,31]. In the present study, the secretory cavities were covered by two or three modified epidermal cells, commonly referred to as overlying cells, which did not appear to form a specialized aperture, as previously observed by Kalachanis and Psaras [27] and Khan et al. [29]. Overlying cells are considered a distinctive anatomical feature in several members of the Myrtaceae family [32], further supporting the taxonomic relevance of these structures. Young stems appear tetrahedral torounded in cross-section, with small ribs., and the secretory cavities were localized under the epidermal tissue and in the outer layers of the cortex parenchyma, as previously reported by Bulavin et al. [33].

Overall, the positive histochemical reactions observed in both leaves and stem fragments confirmed that the pruning residues still retained lipophilic material compatible with EO accumulation, supporting their suitability for distillation and valorization.

From a chemical point of view, both EOs were dominated by oxygenated monoterpenes, with 1,8-cineole as the main compound in both *L. nobilis* and *M. communis* EOs. This finding is consistent with the known chemical profile of laurel EO, in which 1,8-cineole is frequently reported among the major constituents, together with monoterpene hydrocarbons and oxygenated monoterpenes [12]. In the present study, *L. nobilis* EO was characterized mainly by 1,8-cineole, sabinene, and linalool, whereas *M. communis* EO contained 1,8-cineole, α-pinene, and myrtenyl acetate as the most abundant compounds. Therefore, the biological effects of the two EOs should be interpreted in relation to their complete chemical profiles rather than solely to the abundance of 1,8-cineole.

The predominance of 1,8-cineole and α-pinene in myrtle EO is also in line with previous reports describing *M. communis* EO as a monoterpene-rich plant–complex with biological activities of potential interest [16,17]. However, the relative abundance of individual compounds may vary widely depending on genotype, plant organ, phenological stage, geographical origin, environmental conditions, and extraction method. This variability reinforces the need for chemical profiling before any biological or applicative evaluation of EOs intended for agricultural use.

The phytotoxicity results confirmed that both EOs exerted species-specific and concentration-dependent effects on seed germination and radicle elongation. This behavior is consistent with the general phytotoxic profile of EOs and their terpenoid constituents, which have been reported to interfere with several physiological processes involved in early seedling development [7,9,10]. Although 1,8-cineole was the predominant constituent of both EOs and may have contributed to the observed effects, their phytotoxicity cannot be attributed to this compound alone. Essential oils are plant–complexes in which major and minor constituents may interact additively, synergistically, or antagonistically, thereby modifying the activity expected from the individual compounds. Reported phytotoxic mechanisms of EOs and terpenoids include disruption of cell membrane integrity and permeability, alteration of water and ion homeostasis, induction of oxidative imbalance through excessive reactive oxygen species production, interference with cell division and mitotic processes, and modulation of phytohormone-regulated germination and seedling development [7,9,10]. These mechanisms may act simultaneously and could contribute to the species- and concentration-dependent responses observed in the present study. However, since no specific mechanistic endpoints were investigated, these interpretations should be regarded as plausible mechanisms rather than experimentally demonstrated modes of action.

In the present study, radicle elongation generally appeared more responsive than germination, although the direction and magnitude of the effect differed according to the plant species and EO tested. This is an important point, because radicle growth is often considered a sensitive endpoint in phytotoxicity assays, reflecting early alterations in water uptake, cell expansion, membrane stability, and oxidative balance.

For *L. nobilis* EO, the most relevant inhibitory effects were observed on *H. vulgare* germination at the highest concentration and on the radicle elongation of *L. multiflorum* and *S. alba*. These data support the hypothesis that laurel EO may interfere with the early developmental stages of selected plant species, although the observed effects were strongly dependent on the species and endpoint evaluated. Previous studies on laurel EO reported modest or species-dependent phytotoxic activity when the EO was tested alone, whereas stronger effects were observed when it was combined with other EOs or when 1,8-cineole was evaluated as a major active component [14,15]. Therefore, the present findings are consistent with the literature in showing that *L. nobilis* EO has phytotoxic potential, but that its practical use would require optimization of dose, formulation, and target selectivity. *M. communis* EO showed more pronounced phytotoxic effects, particularly against *S. alba*, which was the most sensitive species in both germination and radicle elongation assays. The marked inhibition observed at the highest concentration suggests that myrtle pruning residues may represent a promising source of EO with bioherbicidal potential. This result is particularly relevant because available data on the phytotoxic activity of myrtle EO are still limited. To the best of the authors’ knowledge, this is the first study to investigate the valorization of *L. nobilis* and *M. communis* pruning residues through a combined anatomical, micromorphological, histochemical, phytochemical, phytotoxic, and ecotoxicological assessment of the recovered EOs. This multidisciplinary framework provides a comprehensive evaluation of both their potential as bio-based weed management agents and the environmental limitations associated with their possible application.

Miloudi et al. [19] reported the contribution of *M. communis* EO within an optimized EO mixture active against *Amaranthus retroflexus*, but the selective effects of myrtle EO alone on crops and weeds remain poorly documented. In the present study, *R. sativus* appeared comparatively tolerant and often showed stimulation of radicle elongation, whereas *S. alba* was strongly inhibited by *M. communis* EO. This divergent response may reflect species-specific sensitivity to the EO plant–complex and highlights the need to evaluate both crop and weed species before proposing EO-based products for weed management.

The stimulatory effects observed at some lower concentrations, particularly on radicle elongation of *R. sativus*, should not be overlooked. Low-dose stimulation followed by inhibition at higher concentrations is frequently observed with plant-derived bioactive compounds and may reflect hormetic-like responses. In the context of bioherbicide development, this phenomenon is particularly relevant because suboptimal concentrations may fail to inhibit target weeds or may even stimulate non-target plant growth. Therefore, dose optimization is essential to define an effective concentration range that inhibits target weeds while minimizing undesirable effects on non-target crops. For several species and endpoints, 50% inhibition was not reached within the tested concentration range; therefore, reliable IC_50_/EC_50_ values could not be calculated without extrapolation beyond the experimental data. Moreover, because EOs are volatile and chemically complex mixtures, their biological activity under practical conditions may differ from that observed in controlled Petri dish assays. Future studies should therefore include formulation strategies able to improve EO stability, persistence, and delivery, together with soil, greenhouse, and field assays conducted using agronomically realistic application rates.

The ecotoxicological results added an essential level of interpretation to the potential use of these EOs as bio-based weed management tools. Although plant-derived products are often perceived as intrinsically safer than synthetic herbicides, their natural origin does not automatically imply environmental safety. EOs are biologically active mixtures, and their possible effects on non-target organisms must be considered before practical application [11]. In the present study, the two aquatic invertebrate models showed markedly different responses. *D. magna* neonates were sensitive to both EOs, with a clear concentration-dependent increase in immobilization and EC_50_ values in the range of approximately 0.12–0.16 mg/mL. Conversely, *A. salina* nauplii did not show a significant reduction in viability across the tested concentration range.

This organism-dependent response indicates that the ecotoxicological profile of laurel and myrtle EOs cannot be generalized from a single model organism. The greater sensitivity of *D. magna* may be related to its freshwater physiology, filter-feeding behavior, and continuous exposure of respiratory and ion-regulating surfaces to dissolved or dispersed constituents in the surrounding medium. By contrast, *A. salina* is adapted to saline and highly variable environments and possesses efficient osmoregulatory and physiological stress-response mechanisms. In addition, the high ionic strength of the saline exposure medium may affect the solubility, dispersion, and effective bioavailability of the relatively hydrophobic EO constituents. Differences in habitat, test medium, uptake routes, developmental stage, and physiological tolerance may therefore have contributed to the contrasting responses observed in the two models. Nevertheless, since toxicokinetic and mechanistic endpoints were not investigated, these factors should be regarded as plausible explanations rather than experimentally demonstrated causes. This interpretation is supported by the meta-analysis of Afonso et al. [34], which showed heterogeneous and sometimes contrasting sensitivity among aquatic organisms exposed to EOs and plant extracts and identified *Daphnia* spp. as suitable models for ecotoxicity assessment, whereas *Artemia* spp. appear more appropriate for preliminary hazard screening. The concentrations tested in the present study were selected to characterize concentration-dependent biological responses under controlled laboratory conditions and should not be interpreted as established agricultural application rates or predicted environmental concentrations. In particular, the phytotoxicity assays involved direct exposure of seeds to EO solutions up to 500 μg/mL in Petri dishes, whereas the ecotoxicological assays used concentrations up to 1 mg/mL as a hazard-screening range suitable for identifying effects and defining concentration–response relationships. The highest ecotoxicological concentration represents a conservative laboratory exposure rather than a concentration expected to occur uniformly in natural aquatic compartments following agricultural application. Actual exposure would depend on the formulation, applied amount, application method, volatilization, degradation, adsorption to soil organic matter, runoff, and subsequent dilution in receiving waters. Since no final formulation, field application rate, or environmental fate data were available, predicted environmental concentrations and field risk could not be determined. Therefore, the observed toxicity to *D. magna* should be interpreted as preliminary hazard information and an early warning signal requiring further exposure assessment, rather than as a direct prediction of toxicity under realistic agricultural conditions.

In this perspective, encapsulation, controlled-release systems, or targeted application methods may help reduce non-target exposure while preserving phytotoxic efficacy against weeds.

Overall, the present study supports the circular valorization of *L. nobilis* and *M. communis* pruning residues as sources of chemically characterized EOs with phytotoxic potential. Among the two EOs, *M. communis* showed the most promising inhibitory activity, particularly against *S. alba*, whereas *L. nobilis* displayed a more moderate and species-dependent profile. At the same time, the sensitivity of *D. magna* emphasizes that the development of EO-based bioherbicides must be accompanied by a careful evaluation of selectivity and environmental safety. Further studies should include formulation development, degradation and persistence assays, soil, greenhouse, and field experiments, evaluation of additional non-target organisms, and comparison with conventional herbicides under environmentally and agronomically realistic exposure scenarios.

## 4. Materials and Methods

### 4.1. Plant Material

Pruning residues of *L. nobilis* and *M. communis* consisted of young current-year branches bearing leaves, collected from established hedges maintained by periodic pruning (Figure S1). The plant material was collected from two different sites in the Campania Region, Southern Italy. Specifically, *L. nobilis* residues were collected from hedges growing in a private field in Agerola, Naples (40.631326° N, 14.548445° E), whereas *M. communis* residues were collected from hedges growing in the gardens of the University of Salerno, Fisciano, Salerno (40.773902° N, 14.786832° E). The plant material was transported to the laboratory in paper bags and air-dried at room temperature before steam distillation. The amounts of dried plant material subjected to distillation were 11.10 kg for *L. nobilis* and 18.12 kg for *M. communis*.

### 4.2. Anatomical and Micromorphological Analyses

Anatomical details of pruning residues, including leaves and young stems of *M. communis* and *L. nobilis,* were analyzed using a Leica DM 2000 fluorescence microscope equipped with an H3 filter (excitation filter BP 420–490 nm) (Leica Microsystems, Wetzlar, Germany) and a ToupCam digital camera (CMOS sensor, 3.1 MP resolution, ToupTek Photonics, Hangzhou, China).

Small fresh portions of the leaf surfaces and handmade transverse sections of plant materials, obtained using a double-edged razor blade, were first cleared with an aqueous solution of chloral hydrate and mounted in a chloral hydrate-glycerol medium [35]. Other sections were then stained with specific histochemical reagents for lipophilic substances, namely Fluorol Yellow 088 and Sudan Black [36], to highlight the presence of EOs.

Micromorphological observations were performed using a VEGA3 Tescan type LMU Scanning Electron Microscope (Tescan USA Inc., Cranberry Twp, PA, USA), operating at an accelerating voltage of 20 kV. For this purpose, small portions of leaves and young stems of both species were then fixed in a Finefix working solution for 24 h at 4 °C, dehydrated through an ethanol series (70, 80, 90, and 100%) for 1 h each [37], and finally critical-point dried using a K850CPD 2M apparatus (K850CPD 2M, Strumenti S.r.l., Rome, Italy). The dried specimens were then mounted on aluminum stubs using adhesive carbon tape and coated with a 10 nm gold layer [38].

### 4.3. Essential Oils Extraction

Pruning material from both plants was subjected to steam distillation for 2 h, following the procedure reported in the European Pharmacopeia [39]. The EOs obtained were subsequently solubilized in n-hexane, filtered over anhydrous sodium sulfate, and subjected to a flow of N_2_ to remove the residual solvent. The EOs were then stored in an amber vial at 4 °C, protected from light, heat, and humidity, until further analysis. The percentage yields were 0.42% for *L. nobilis* and 0.26% for *M. communis*.

### 4.4. Gas Chromatography with Flame Ionization Detection (GC-FID) and Gas Chromatography–Mass Spectrometry (GC-MS) Analyses

The chemical characterization of the EOs was performed by GC-FID and GC-MS analyses. GC-FID determinations were performed using a Perkin-Elmer Sigma 115 gas chromatograph (Waltham, MA, USA) equipped with a low-polarity fused silica HP-5MS capillary column (30 m × 0.25 mm i.d.; 0.25 μm film thickness). GC-MS analyses were performed using an Agilent 6850 Series II system (Agilent, Santa Clara, CA, USA), equipped with an HP-5MS fused silica capillary column (Agilent, 30 m × 0.25 mm i.d.; 0.25 μm film thickness) and coupled to an Agilent 5973 mass spectrometer. Spectra were acquired in electron impact ionization (EI) mode at 70 eV, with the ion multiplier voltage set to 2000 V. Mass spectra were recorded in the *m*/*z* range of 50–500, with an acquisition rate of five scans per second. GC-FID and GC-MS analyses were performed using identical chromatographic conditions. The injector temperature was maintained at 250 °C; the FID detector was set to 290 °C, while the mass spectrometer quadrupole was maintained at 150 °C. The oven thermal program included an initial isothermal phase at 40 °C for 5 min, followed by a temperature increase of 2 °C/min until reaching 270 °C, and a subsequent isothermal hold at that temperature for 20 min. To confirm the identification of the constituents, the analyses were also repeated using an HP Innowax polar column (50 m × 0.20 mm i.d.; 0.25 μm film thickness), keeping the operating parameters unchanged. Helium was used as the carrier gas with a constant flow rate of 1.0 mL/min. The identification of the compounds was carried out by comparing the Kovats retention indices (KI) with values reported in the literature [40,41,42,43], as well as by comparing the mass spectra obtained with those contained in the NIST 17 and Wiley 275 libraries [44]. The Kovats indices were calculated using a homologous series of n-alkanes between C10 and C35, analyzed under the same experimental conditions. The relative percentages of the individual components were determined by normalizing the areas of the chromatographic peaks, without applying response correction factors.

### 4.5. Phytotoxic Assay

Phytotoxic effects were assessed by examining the germination and radicle elongation of seeds of two plants of agricultural interest, *R. sativus* (radish) and *H. vulgare* (barley), as well as two weeds, *L. multiflorum* (Italian ryegrass) and *S. alba* (wild mustard). Seeds of *R. sativus* and *H. vulgare* were purchased from Blumen group s.r.l., Bologna, Italy, while seeds of *L. multiflorum* were obtained from Fratelli Ingegnoli s.p.a., Milan, Italy. Seeds of *S. alba* were collected from wild populations. These seeds are commonly used in phytotoxicity assessments due to their ease of germination and well-documented germination behavior. Before testing, seeds were sterilized with 95% ethanol for 15 s and then placed in Petri dishes (Ø 90 mm) on three layers of Whatman filter paper, soaked with 7 mL of distilled water for the preliminary germination test, or with 7 mL of the test solutions for the phytotoxicity assay. To improve solubility, the EO was dissolved in a mixture of water and acetone (99.5:0.5 *v*/*v*) and tested at concentrations of 500, 250, 125, and 63 μg/mL. The same water/acetone mixture without EO was used as the negative control. Germination conditions were maintained at 20 ± 1 °C under a natural photoperiod. The seeds were first tested in water only to evaluate their effective germination. The progress of seed germination was monitored in Petri dishes at 24 h intervals, with a seed considered germinated when radicle protrusion became visible [45]. After 120 h for *R. sativus*, *S. alba*, and *H. vulgare* and 168 h for *L. multiflorum*, germination was observed, and radicle lengths were measured in cm. Each measurement was performed in triplicate, using Petri dishes containing 10 seeds each.

### 4.6. Ecotoxicological Assays

The potential environmental hazard of the tested essential oils was evaluated using two aquatic invertebrate models representative of two different ecosystems: *Artemia salina* nauplii, a marine/brackish-water model, and *Daphnia magna* neonates, a freshwater model. Toxic effects were expressed as the percentage of viable nauplii in the *A. salina* assay and as the percentage of immobilized organisms in the *D. magna* assay. Data are presented as mean ± standard deviation (SD) of three independent experiments. Within each experiment, each concentration was tested in triplicate for *A. salina* and in quadruplicate for *D. magna* according to Malaspina et al. [22].

#### 4.6.1. *Daphnia magna* Toxicity Assay

Freshwater ecotoxicity was investigated using neonates of *Daphnia magna*, obtained from the commercial DAPHTOXKIT F. Dormant eggs were hatched in Petri dishes under continuous illumination and aeration using a standard freshwater medium, which was prepared from distilled water supplemented with NaHCO_3_, CaCl_2_·2H_2_O, MgSO_4_·7H_2_O, and KCl. The hatching medium was aerated before use. Hatching occurred within 48–72 h, and neonates were used for the toxicity assay within 24 h of hatching. Working solutions of the EOs were prepared by diluting DMSO stock solutions in the same freshwater medium used for hatching, to achieve final concentrations ranging from 1 to 0.03125 mg/mL. The final DMSO concentration was maintained at 0.1% in all test solutions and controls. Each concentration was tested in four replicate wells, each containing five neonates. Freshwater containing 0.1% DMSO served as the negative control, whereas potassium dichromate (K_2_Cr_2_O_7_, 3 μg/mL) was included as the positive control. Plates were sealed with Parafilm and incubated in the dark at RT. Immobilization was recorded after 24 and 48 h using a stereomicroscope. Individuals that showed no movement within 15 s of observation, even after gentle agitation, were considered immobile.

#### 4.6.2. *Artemia salina* Toxicity Assay

Marine ecotoxicity was evaluated using hatched *Artemia salina* nauplii. Cysts were incubated in artificial seawater at 3% salinity, prepared by dissolving 33.33 g of sea salt in 1 L of tap water, under continuous aeration and constant illumination at RT for 36–48 h. Immediately after hatching, nauplii were used for the assay.

Essential oils were first dissolved in DMSO and then diluted in artificial seawater in order to obtain final concentrations of 1, 0.5, 0.25, 0.125, 0.0625, and 0.03125 mg/mL. The final DMSO concentration was maintained at 0.1% in all test solutions and controls. Ten nauplii were transferred into each well of a 24-well plate containing 2 mL of the corresponding test solution. Artificial seawater supplemented with 0.1% DMSO was used as the negative control, whereas potassium dichromate (K_2_Cr_2_O_7_, 50 μg/mL) served as the positive control. Following 24 and 48 h of exposure at RT, organism viability was assessed under a stereomicroscope. Nauplii were considered non-viable when no spontaneous movement or response to gentle mechanical stimulation was observed.

### 4.7. Statistical Analysis

Statistical analysis of phytotoxic activity and ecotoxicological assays was performed by analysis of variance (ANOVA) using GraphPad Prism 6.0 (GraphPad Software, San Diego, CA, USA). The results were compared with the corresponding negative control and considered statistically significant (*p* < 0.05) by Dunnett’s multiple-comparison test. For ecotoxicological assays, concentration–response curves were generated when appropriate, and EC_50_ values with 95% confidence limits were calculated using nonlinear regression analysis. Data are expressed as mean ± standard deviation (SD) of three independent experiments.

## 5. Conclusions

This study supports the circular valorization of *Laurus nobilis* and *Myrtus communis* pruning residues as sustainable sources of chemically characterized essential oils. Anatomical and histochemical analyses confirmed the presence of secretory structures still rich in lipophilic compounds, while GC-FID and GC-MS profiling showed that both EOs were dominated by oxygenated monoterpenes, with 1,8-cineole as the main constituent.

Both EOs showed species- and concentration-dependent phytotoxic effects, with *M. communis* EO displaying the most promising inhibitory activity, particularly against *Sinapis alba*. However, the ecotoxicological assays revealed an organism-dependent response, with limited effects on *Artemia salina* but marked concentration-dependent immobilization of *Daphnia magna*. These findings indicate that laurel and myrtle pruning residues may represent valuable bioresources for the development of EO-based weed management tools, but also highlight the need for further studies on formulation, selectivity, environmental fate, and non-target toxicity before practical agricultural application.

## Figures and Tables

**Table 1:** Chemical composition of *L. nobilis* essential oil.

N.	Compound	%	Ki ^a^	Ki ^b^	Identification ^c^
1	α-Thujene	0.5	928	1027	1, 2
2	α-Pinene	6.96	934	1025	1, 2, 3
3	Camphene	0.6	945	1068	1, 2, 3
4	Sabinene	16.05	973	1122	1, 2
5	β-Myrcene	1.71	993	1161	1, 2
6	1,8-Cineole	46.31	1030	1211	1, 2, 3
7	γ-Terpinene	0.73	1056	1245	1, 2, 3
8	Linalool	13.67	1099	1543	1, 2, 3
9	Terpinen-4-ol	1.08	1174	1601	1, 2
10	α-Terpineol	2.31	1190	1694	1, 2
11	β-Terpinyl acetate	5.68	1349	1622	1, 2
12	Eugenol	0.99	1356	2163	1, 2
13	Methyleugenol	3.41	1405	2006	1, 2
	Total	100			
	Hydrocarbon monoterpenes	26.55			
	Oxygenated monoterpenes	69.05			
	Phenylpropanoids	4.40			

^a,b^ Kovats retention indices determined relative to a series of n-alkanes (C10–C35) on the apolar HP-5 MS and the polar HP Innowax capillary columns, respectively. ^c^ Identification method: 1 = comparison of the Kovats retention indices with published data, 2 = comparison of mass spectra with those listed in the NIST 17 and Wiley 275 libraries and with published data; 3 = co-injection with authentic compounds.

**Table 2:** Chemical composition of *M. communis* essential oil.

N.	Compound	%	KI ^a^	KI ^b^	Identification ^c^
1	α-Thujene	0.44	928	1027	1, 2
2	α-Pinene	18.43	934	1025	1, 2, 3
3	β-Pinene	0.31	972	1111	1, 2, 3
4	β-Myrcene	0.34	993	1161	1, 2
5	α-Phellandrene	0.48	999	1165	1, 2
6	3-Carene	0.43	1004	1150	1, 2
7	p-Cymene	0.31	1021	1278	1, 2
8	1,8-Cineole	41.53	1030	1211	1, 2, 3
9	cis-β-Ocimene	0.91	1048	1242	1, 2
10	γ-Terpinene	0.76	1056	1245	1, 2, 3
11	Terpinolene	0.70	1085	1279	1, 2
12	Linalool	8.51	1099	1543	1, 2, 3
13	α-Terpineol	2.28	1190	1694	1, 2
14	Estragole	0.45	1197	1671	1, 2, 3
15	Linalyl acetate	2.29	1257	1543	1, 2
16	Myrtenyl acetate	16.06	1323	1698	1, 2
17	α-Terpinyl acetate	0.97	1347	1709	1, 2
18	Geranyl acetate	3.08	1387	1764	1, 2
19	Methyleugenol	0.65	1405	2006	1, 2
20	β-Caryophyllene	0.48	1409	1607	1, 2
21	Humulene	0.59	1446	1672	1, 2
	Total	100			
	Hydrocarbon monoterpenes	23.11			
	Oxygenated monoterpenes	74.72			
	Phenylpropanoids	1.10			
	Hydrocarbon sesquiterpenes	1.07			

^a,b^ Kovats retention indices determined relative to a series of *n*-alkanes (C_10_–C_35_) on the apolar HP-5 MS and the polar HP Innowax capillary columns, respectively. ^c^ Identification method: 1 = comparison of the Kovats retention indices with published data, 2 = comparison of mass spectra with those listed in the NIST 17 and Wiley 275 libraries and with published data; 3 = co-injection with authentic compounds.

**Table 3:** Phytotoxic activity of *L. nobilis* EO.

Number of Germinated Seeds				
	*Hordeum vulgare*	*Raphanus sativus*	*Lolium multiflorum*	*Sinapis alba*
Control H_2_O+C_3_H_6_O	5.6 ± 0.6 (0.0)	6.7 ± 2.1 (0.0)	8.7 ± 0.6 (0.0)	10.0 ± 0.0 (0.0)
Treatment (µg/mL)				
63	5.0 ± 1.0 *** (10.7)	6.3 ± 1.2 *** (6.0)	6.7 ± 0.6 **** (23.0)	10.0 ± 0.0 (0.0)
125	5.3 ± 1.1 *** (5.4)	6.7 ± 1.2 ** (0.0)	7.0 ± 1.0 **** (19.5)	9.3 ± 0.6 (7.0)
250	5.0 ± 1.1 *** (10.7)	5.3 ± 1.5 *** (20.9)	8.0 ± 2.0 (8.0)	9.3 ± 0.6 (7.0)
500	1.3 ± 0.6 * (76.8)	4.7 ± 1.5 ** (30.0)	7.3 ± 1.5 * (16.1)	7.7 ± 1.2 (23.0)
**Radicle length (cm)**				
	*Hordeum vulgare*	*Raphanus sativus*	*Lolium multiflorum*	*Sinapis alba*
Control H_2_O+C_3_H_6_O	2.4 ± 1.0 (0.0)	1.5 ± 0.8 (0.0)	3.3 ± 1.0 (0.0)	2.4 ± 0.9 (0.0)
Treatment (µg/mL)				
63	2.4 ± 1.0 *** (0.0)	4.0 ± 2.9 (−166.7)	3.6 ± 1.3 * (−9.1)	2.6 ± 1.1 *** (−8.3)
125	2.1 ± 0.5 *** (12.5)	1.6 ± 0.8 ** (−6.7)	3.6 ± 1.1 *** (−9.1)	2.5 ± 1.0 *** (−4.2)
250	2.8 ± 1.0 *** (−16.7)	2.5 ± 1.2 ** (−66.7)	3.8 ± 1.5 *** (−15.2)	1.6 ± 1.0 **** (33.3)
500	1.5 ± 1.8 * (37.5)	2.4 ± 1.8 ** (−60.0)	1.9 ± 0.9 *** (42.4)	1.5 ± 1.2 **** (37.5)

The results are expressed as the mean of three experiments ± SD. The percentage of inhibition is reported in round brackets. * *p* < 0.05; ** *p* < 0.01; *** *p* < 0.001; **** *p* < 0.0001 compared with control (ANOVA followed by Dunnett’s multiple-comparison test).

**Table 4:** Phytotoxic activity of *M. communis* EO.

Number of Germinated Seeds				
	*Hordeum vulgare*	*Raphanus sativus*	*Lolium multiflorum*	*Sinapis alba*
Control H_2_O+C_3_H_6_O	5.7 ± 0.6 (0.0)	6.0 ± 1.0 (0.0)	9.7 ± 0.6 (0.0)	9.3 ± 0.6 (0.0)
Treatment (µg/mL)				
63	5.7 ± 0.6 *** (0.0)	6.3 ± 0.6 ** (−5.0)	6.0 ± 1.0 *** (38.1)	9.0 ± 1.0 ** (3.2)
125	4.0 ± 1.0 *** (29.8)	6.0 ± 1.7 (0.0)	7.0 ± 2.6 (27.8)	9.0 ± 1.0 ** (3.2)
250	4.7 ± 2.5 (17.6)	8.0 ± 2.0 (−33.3)	8.0 ± 1.7 (17.5)	6.3 ± 0.6 *** (32.3)
500	3.3 ± 2.1 (42.1)	5.3 ± 0.6 **** (11.7)	7.7 ± 1.2 * (20.6)	2.0 ± 1.0 ** (78.5)
**Radicle length (cm)**				
	*Hordeum vulgare*	*Raphanus sativus*	*Lolium multiflorum*	*Sinapis alba*
Control H_2_O+C_3_H_6_O	0.0	0.0	0.0	0.0
Treatment (µg/mL)				
63	4.0 ± 1.4 ** (−48.1)	1.6 ± 0.7 ***(−33.3)	4.0 ± 1.6 *** (−17.6)	1.7 ± 0.8 **** (−13.3)
125	2.4 ± 0.7 ** (11.1)	2.1 ± 0.7 *** (−75)	3.3 ± 1.2 *** (2.9)	1.5 ± 0.8 **** (0.0)
250	2.3 ± 1.4 ** (14.8)	1.6 ± 0.7 *** (−33.3)	3.8 ± 1.2 *** (−11.7)	0.8 ± 0.3 **** (46.7)
500	1.6 ± 0.9 *** (40.8)	1.7 ± 0.5 *** (−41.7)	2.6 ± 0.7 **** (23.5)	0.3 ± 0.3 **** (80.0)

The results are expressed as the mean of three experiments ± SD. The percentage of inhibition is reported in round brackets. * *p* < 0.05; ** *p* < 0.01; *** *p* < 0.001; **** *p* < 0.0001 compared with control (ANOVA followed by Dunnett’s multiple-comparison test).

## Data Availability

The original contributions presented in this study are included in the article. Further inquiries can be directed to the corresponding authors.

## Supplementary Materials

The following supporting information can be downloaded at: https://www.mdpi.com/article/10.3390/molecules31162741/s1. Figure S1. Bay laurel (A) and myrtle (B) hedges. Arrows indicate the young current-year branches periodically pruned to maintain the geometric shape of the hedges and used as plant material in the present study.

## Abbreviations

The following abbreviations are used in this manuscript:

ANOVA	Analysis of variance
CL	Confidence limits
CTR−	Negative control
CTR+	Positive control
DMSO	Dimethyl sulfoxide
EC_50_	Median effective concentration
EI	Electron impact ionization
EO	Essential oil
EOs	Essential oils
FID	Flame ionization detection
GC	Gas chromatography
GC–FID	Gas chromatography with flame ionization detection
GC–MS	Gas chromatography–mass spectrometry
KI	Kovats retention index
LM	Light microscopy
MAPs	Medicinal and aromatic plants
MS	Mass spectrometry
NIST	National Institute of Standards and Technology
SD	Standard deviation
SEM	Scanning electron microscopy
