# Supplementary material for: Circular Valorization of Laurel and Myrtle Pruning Residues: Chemical Profiling, Phytotoxicity, and Ecotoxicological Assessment of Essential Oils for Sustainable Weed Management
Source: Molecules. 2026 Aug 7;31(16):2741. doi: 10.3390/molecules31162741 (PMC13515649; doi:10.3390/molecules31162741)
Supplement: Supplementary file 1 [file molecules-31-02741-s001.zip › molecules-4454465-supplementary.pdf]

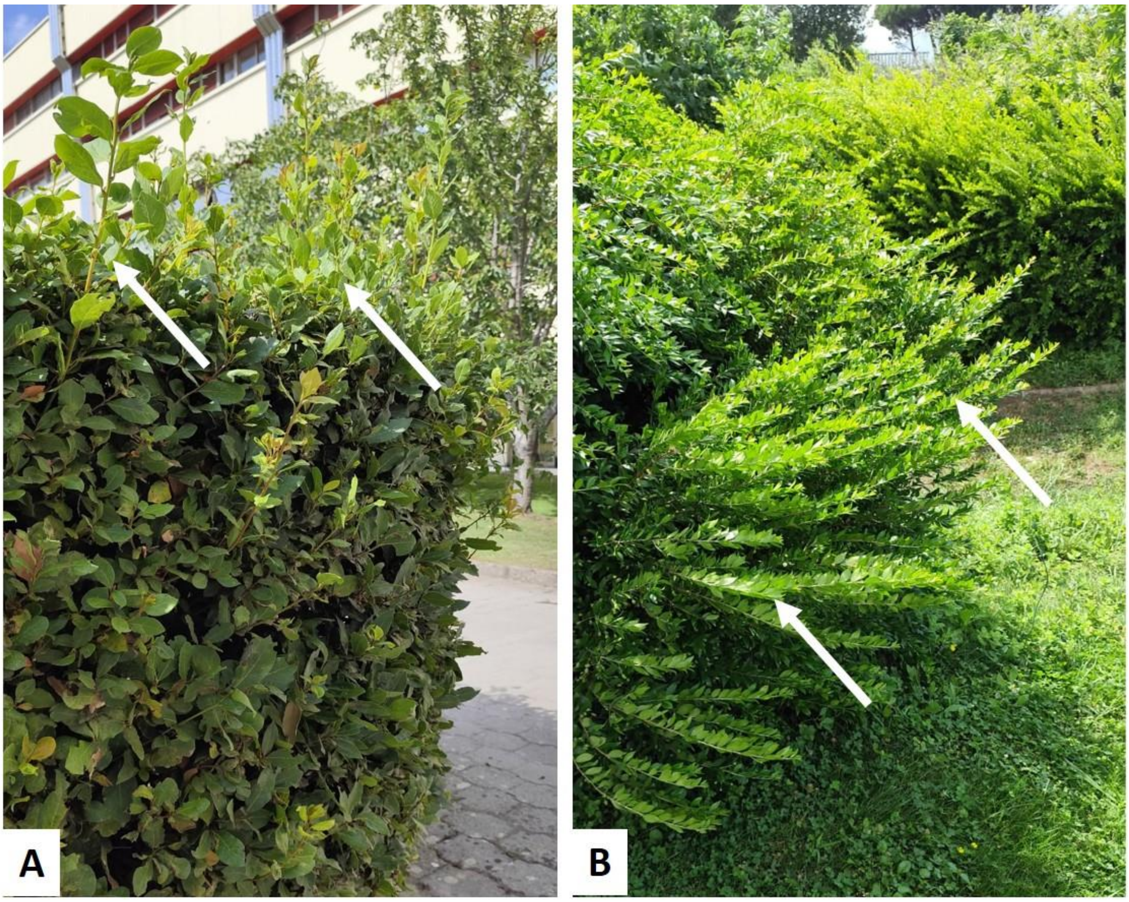

Figure S1. Bay laurel (A) and myrtle (B) hedges. Arrows indicate the young current-year branches periodically pruned to maintain the geometric shape of the hedges and used as plant material in the present study.
